# Spina Bifida

**Published:** 1861-08

**Authors:** 

**Affiliations:** Chicago


ARTICLE III.

### SPINA BIFIDA.—BY VINDEX.

TO THE EDITORS OF THE CHICAGO MEDICAL EXAMINER.

GENTLEMEN:—A perusal of the periodical literature of our profession cannot fail occasionally to impress the reader with the great anxiety some manifest to secure a niche in the temple of Fame, be it never so humble, provided they can thus enjoy a transient glance from their fellows, or perhaps even reach a fleeting and ephemeral notoriety.

This longing for distinction exhibits itself in various ways. At times it is seen in the modification of instruments, often termed "improvement" of such, when the very reverse, through the ingenuity of the inventor, is effected. Then, again, we have "improved" modes of operation, wherein some unimportant innovation is proposed. Or, it may be, that a master-mind has suggested a relation between a particular remedy and a disease; the suggestion, in itself, involving all the originality of discovery, and all the merit of subsequent development. Still, as we know, science is slow in coming to maturity, and time may be required for a full unfolding of the germ in the perfection of ultimate practical ntility. In the interval, however, some aspirants after fame will be occasionally found endeavoring to anticipate the subsequent efforts of the author, who, having supplied them with a clew, has thus facilitated their task. But not content with quartering their own empty performances with his substantial merit, they will not hesitate to thrust themselves prominently forward, and by this *modest* intervention, try to eclipse his well-earned fame. These reflections have been suggested by a glance at the Report, made by the Chairman of the Committee of Surgery, presented at the Tenth Annual Meeting of the Illinois State Medical Society. Section xviii. of this "Report" relates to the treatment of spina bifida by injection of iodine. The reporter tells us that this operation was first proposed and performed in Chicago, May 3, 1848. In the Report we are not directly informed by whom it was, at that time, proposed and performed; but from its scattered and fragmentary materials we are permitted indirectly to gather the claim of the reporter himself to originality of planning and priority of performing the operation in question. Now, I am not disposed to recognize either claim. So far back as 1836, Velpeau employed iodine as an injection in hydrocele with such success as to extend its application to *various serous cysts.* In his "Operative Medicine," published in 1839, he suggested it might, without danger, be even thrown into the peritoneal cavity; and in 1840, he thus injected the knee joint. In injecting such a tumour as that of spina bifida, no one supposes that he deals with anything more than a serous cyst, as far, at least, as the action of the iodine on its surface is concerned. He does not imagine he thus remedies an organic affection of the nervous tissue, that may be at the time associated with it. If the disease be accompanied by this morbid condition it is not susceptible of remedy from any plan of treatment; if otherwise, it is to be considered a serous cyst, and to be treated as such. The humblest intellect might conclude that similarity of structure and of diseased action necessarily indicate the suitableness of similar therapeutic means. In fact, as Velpeau himself remarks, injection succeeds best in proportion as the cavity more nearly approaches a simple serous one. Thus, in cysts of the neck, where the membrane containing the fluid is everywhere surrounded by soft tissues, the operation never fails. It is to Velpeau, then, that science is indebted for suggesting this mode of treatment in serous cysts, and their allied diseases. The conception was the fruit of his understanding, and of his only. That the reporter's claim to having first performed the operation is not recognized in France by the profession, the affirmative of which we might be induced to infer by reading the *Chicago Medical Journal*, I beg to refer your readers to a review of the *Bulletin de Thérapeutique*, by the editor of the *Gazette Médicale* of April, 1854.* The reviewer there tells us distinctly that Velpeau was the first who performed the operation of injecting the sac of spina bifida. On the same occasion he notices Chassaignac's case, to which allusion is made in the report before me. Now, if the claim of the writer of the report had been allowed in France, is it probable that the accomplished editor of the *Gazette Médicale* would have told us that Chassaignac's was the second example of spina bifida treated by injection of iodine, and that Velpeau's was the first? It was my intention to confine myself to the report and not (to use a legal phrase) "travel out of the record," had not the reporter referred us to the *Chicago Medical Journal* for Sept., 1859. Now, in the same number of this journal I observe, what appears to me, another inaccuracy. The writer informs us that Mott's case of spina bifida, cured by excision, was "probably not connected with the spine." Mott cured two cases by exsection, in one of which he distinctly tells us, that in extirpating the tumour, "a communication of about the size of a crow-quill was found leading into the cavity of the spinal marrow."†

The reporter favors us with some rules to guide us in the operation, the most of which have reference to the selection of a suitable point for the insertion of the trochar, and the restricting of the amount of serum evacuated, to a correspond-

---

* *Vide Gazette Medicale.* April 15, 1854. p. 220.

† *Vide* Velpeau's "New Elements of Operative Surgery," with notes, &c., by Val. Mott. Vol. iii., p. 810.

ing quantity of the fluid to be injected. He then adds: "in the cases operated on by Chassaignac, Nelaton and Velpeau these precautions were not taken." How does the reporter know that these precautions were not taken by Chassaignac and Velpeau? He acknowledges, in the *Chicago Medical Journal* for Sept., 1859, that he had not seen any published observations of Velpeau's case, and it may therefore be presumed he was unacquainted with the details. As to Chassaignac's case, it lies before me, and I see nothing in it to warrant such an observation. Both were successful. Does the writer mean it should be inferred that, owing to the want of proper precautions, they were not? If this be not his meaning, I cannot avoid remarking, the sentence is most infelicitously expressed.

VINDEX.

CHICAGO, JULY 20, 1861.

